# Surface Modification of Lignite with Alkyl and Mixed Alkyl‐Aryl Films Generated from an Aryl Diazonium Salt and Alkyl Halides: Experimental Results and Theoretical Analyses

**DOI:** 10.1002/open.202300134

**Published:** 2023-12-05

**Authors:** Msc. Gentiana Hasani, Avni Berisha, Dardan Hetemi., Philippe Decorse, Jean Pinson, Fetah I. Podvorica

**Affiliations:** ^1^ Chemistry Department University of Prishtina 10000 Prishtina Kosovo; ^2^ Department of Pharmacy University of Prishtina 10000 Prishtina Kosovo; ^3^ Université Paris Cité, CNRS, ITODYS 75013 Paris France; ^4^ Academy of Sciences and Arts of Kosova, Rr. “Agim Ramadani” Nr. 305 10000 Prishtina Kosovo; ^5^ NanoAlb-Unit of Albanian Nanoscience and Nanotechnology 1000 Tirana Albania

**Keywords:** Alkyl halides, aryl diazonium salts, lignite, surface modification, theoretical modeling

## Abstract

In search of new possible uses of cheap lignite from the Kosova Bassin, the surface of lignite powders is modified with alkyl or mixed alkyl‐aryl layers. Modification is performed in aqueous acid solution containing an aryl diazonium salt and an alkyl halide compound in millimolar concentration, in the presence of potassium iodide as a reducing agent at equimolar concentration. Attachment of alkyl films substituted with carboxylic groups and aryl films with nitro or bis‐trifluoromethyl groups is characterized by IRATR and XPS spectroscopy. The formation of a stable interface during the grafting reactions of alkyl and aryl moieties with lignite surface has been confirmed by theoretical calculations. Aryl diazonium salts once chemically or spontaneously reduced are a source of aryl radicals, able to attach chemically to the material surface or to react with alkyl halides by abstracting the halogen atom. If the aryl diazonium salts are unable to graft to the coal surface due to steric hindrance, they can, nevertheless, abstract an iodine or bromine atom to generate alkyl radicals that react with the material surface.

## Graphical abstract

The surface of lignite has been modified with alkylcarboxylic moieties derived from alkyl halides by diverting the reactivity of the sterically hindered aryl radical obtained by reduction of the 2,6‐dimethylbenzene diazonium tetrafluoroborate after its chemical reduction. Mixed alkyl‐aryl layers are prepared by using 4‐nitro or 3,5‐bis‐trifluoro benzenediazonium salts.

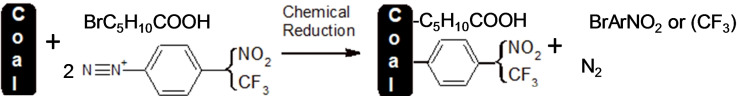

## Introduction

Carbons are used in many domains such as material science, engineering and nanotechnology due to their electrical, mechanical, chemical properties, thermal stability, possible high surface area, ease of handling etc.[^1^, ^2^] Lignite is a heterogeneous material that contain the lowest carbon percentage among various types of coal (25‐35 %). It is so cheap that it is common to build lignite‐fired power plants adjacent to lignite mines in order to limit the cost of transportation, for this reason there is no free market price formation. Lignite has a low calorific power and produces ashes (from 14 to 17 %) containing trace elements such as Co, Cr, Ni, Zn.^[3]^ Therefore, burning lignite has a high environmental impact, this is why it is of interest look for other applications. It has already been tested as adsorbent for removal of many pollutants or as a filler for the preparation of heterogeneous asymmetric reverse osmosis membranes.[^4^, ^5^]

Aryl diazonium salts are widely used reagents for the attachment of organic moieties onto metal, semiconductor, polymer and carbon surfaces including coal, as heterogeneous material by electrografting or chemical reduction.[^6^, ^7^, ^8^] These compounds are easily prepared in aqueous and organic solvents from a variety of commercially available primary aryl amines and the grafting process is performed within short time laps. Aryl diazonium salts, upon electrochemical reduction, give very reactive aryl radicals that react immediately with the carbon electrode leading to the attachment of covalently bonded aryl layers.^[6]^ The reduction of aryl diazonium salts is also performed photochemically or thermally and permitted the successful modification of carbon materials with aromatic films.[^9^, ^10^, ^11^] Whatever the method used for the reduction of aryl diazonium salts, the resulting aryl layers are covalently bonded to the carbon surface[^12^, ^13^] and resist temperatures higher than 400 °C.^[11]^ Nanocomposite materials made of carbon and aryl layers are used for many purposes such as: sensors and biosensors[^14^, ^15^, ^16^] molecular electronics,^[17]^ energy storage,^[18]^ to tune the conductivity of graphene,^[19]^ to improve separation efficacy of heterogenous asymmetric membranes made of cellulose acetate‐lignite etc.^[8]^

However, the use of same approach by use of alkyldiazoniums salts to graft alkyl groups to carbonaceous surfaces is not achievable because these compounds are very unstable and decompose instantaneously once synthesized.^[20]^

Alkyl halides constitute another category of compounds susceptible to give surface bonded groups by electrochemical reduction at very negative potential, that is, at high driving force, in organic solvents. This makes their direct electrografting onto carbon surface less efficient; during their electrochemical reduction alkyl anions (that do not react with surfaces) are produced.^[21]^ 2,6‐Dimethylbenzenediazonium salt (2,6‐DMBD) is electrochemically reduced at the same potential as other aryl diazonium salts but does not react with surfaces due to the steric hindrance of the resulting 2,6‐dimethylphenyl radical.^[12]^ However, we have shown previously the 2,6‐dimethylphenyl radicals abstracts halide atoms from alkyl halides leading to alkyl radicals that attach to carbon and gold surfaces.^[22]^ The use of this crossover reaction permits the generation of alkyl radicals under quite mild conditions at the difference of the direct grafting of alkyl halides.^[23]^ The generation of alkyl radicals by diverting the reaction of aryl radicals with alkyl halides has also been performed with other aryl diazoniums, in this case mixed alkyl aryl layers were attached to glassy carbon surfaces.^[24]^

**Scheme 1:**
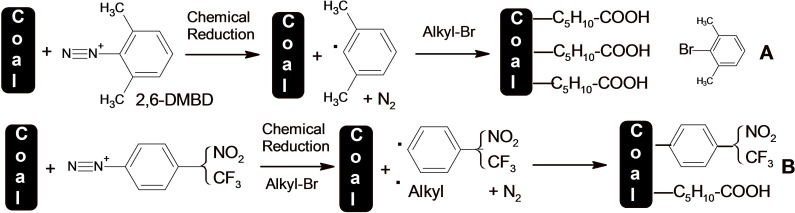
A) Chemical reduction of 2,6‐DMBD and the formation of alkyl radicals that attach to the lignite surface; B) Reduction of an aryldiazonium salt without steric hindrance i) leads to aryl radicals and ii) also create alkyl radicals, Alkyl⋅, both radicals attack the lignite surface to give a mixed layer.

In this paper we introduce alkyl groups on the surface of lignite via a crossover reaction initiated by aryl radicals generated from different aryl diazonium including 2,6‐DMBD. The reaction is performed for the first time on lignite surfaces, in aqueous acid solution. The modified surfaces are characterized by IRATR and XPS. Scheme 1 shows the principles of surface modification with alkyl or mixed alkyl–aryl layers.

## Experimental Section

**Chemicals**: 6‐bromohexanoic acid, (97 %), 2,6‐dimethylaniline, (99 %), sodium nitrite (97 %), 2‐bromoethanoic acid (97 %), 4‐iodobutanoic acid (97 %), 4‐nitrobenzenediazonium tetrafluoroborate (97 %), potassium iodide (97 %) and 37 % solution of hydrochloric acid were purchased from Sigma Aldrich. 3,5‐bis‐trifluoromethylbenzenediazonium tetrafluoroborate (CF_3_)_2_C_6_H_3_BF_4_ is prepared in situ according to the procedure used elsewhere.^[12]^

**Infrared Attenuated Total Reflection Spectroscopy** (ATR) spectra of the modified lignites were recorded using a purged (low CO_2_, dry air) Jasco FT/IR‐6100 Fourier transform IR spectrometer equipped with a germanium ATR accessory (Jasco ATRPR0470‐H). For each spectrum, 1000 scans were accumulated with a spectral resolution of 4 cm^−1^. The background recorded before each spectrum was that of a native (unmodified) lignite substrate.

**XPS** spectra measurements were performed using a K Alpha+ system (Thermo Fisher Scientific, East‐Grinstead, UK) fitted with a micro focused and monochromatic Al Kα X‐ray source (1486.6 eV, spot size: 400 mm). The spectra were calibrated against the C−C/C−H C1 s component set at 285 eV. The chemical composition was determined with version 5.9902 Avantage software, by using the manufacturer sensitivity factors.

**SEM images** have been recorded on a ZEISS Gemini SEM 360 microscope.

**TGA‐GC‐MS**. Thermogravimetric analyses were performed using a Setaram Labsysevo under helium from room temperature to 800 °C with a temperature ramp of 10 K min^−1^. The FTIR spectra of evolved gaseous products during TG analysis were recorded using a Thermoscientific Is 10 infrared spectrometer, and gaseous products evolved were also analyzed using a GC‐MS Thermoscientific Trace1310ISQ‐QD.

**Theoretical calculations**. The DMol3 module of the Materials Studio application was used to optimize the structures. The calculations were performed at GGA level of theory, using DNP polarization basis set^[25]^ in conjunction with the PBE functional.^[26]^ The van der Waals interaction in the calculations were included via Tkatchenko‐Scheffler method.^[27]^ For the field to converge in a self‐consistent approach, there must be an energy difference of less than 10^−7^ Ha. In the calculations of DFT, the conductor‐like screening model, also known as COnductor‐like Screening MOdel (COSMO), was used to find an explanation for the solvent‐water interaction.[^28^, ^29^, ^30^]

**Lignite modification**. Lignite modification in aqueous solution was performed as follows: the lignite specimens were treated with boiling water under stirring to remove all soluble materials, i. e. inorganic part, color, etc. The reaction mixture was filtered with a 589 Blue ribbon and then washed by successive aliquots of water. Finally, the modified lignite was dried at 105 °C to constant weight, grounded and sieved. The lignite fractions of sieve size of ≈170 mesh were used in this study. Typically, 8 g of this lignite were dispersed in 50 mL 0.5 M HCl+an aryl diazonium salt isolated or prepared in situ to a final 20 mM concentration+potassium iodide of 20 mM + 20 mM of an alkyl halide at room temperature for 1 h. The solution was vigorously mixed by ultrasonication or by stirring.

## Results and Discussion

### Unmodified Lignite

**Figure 1:**
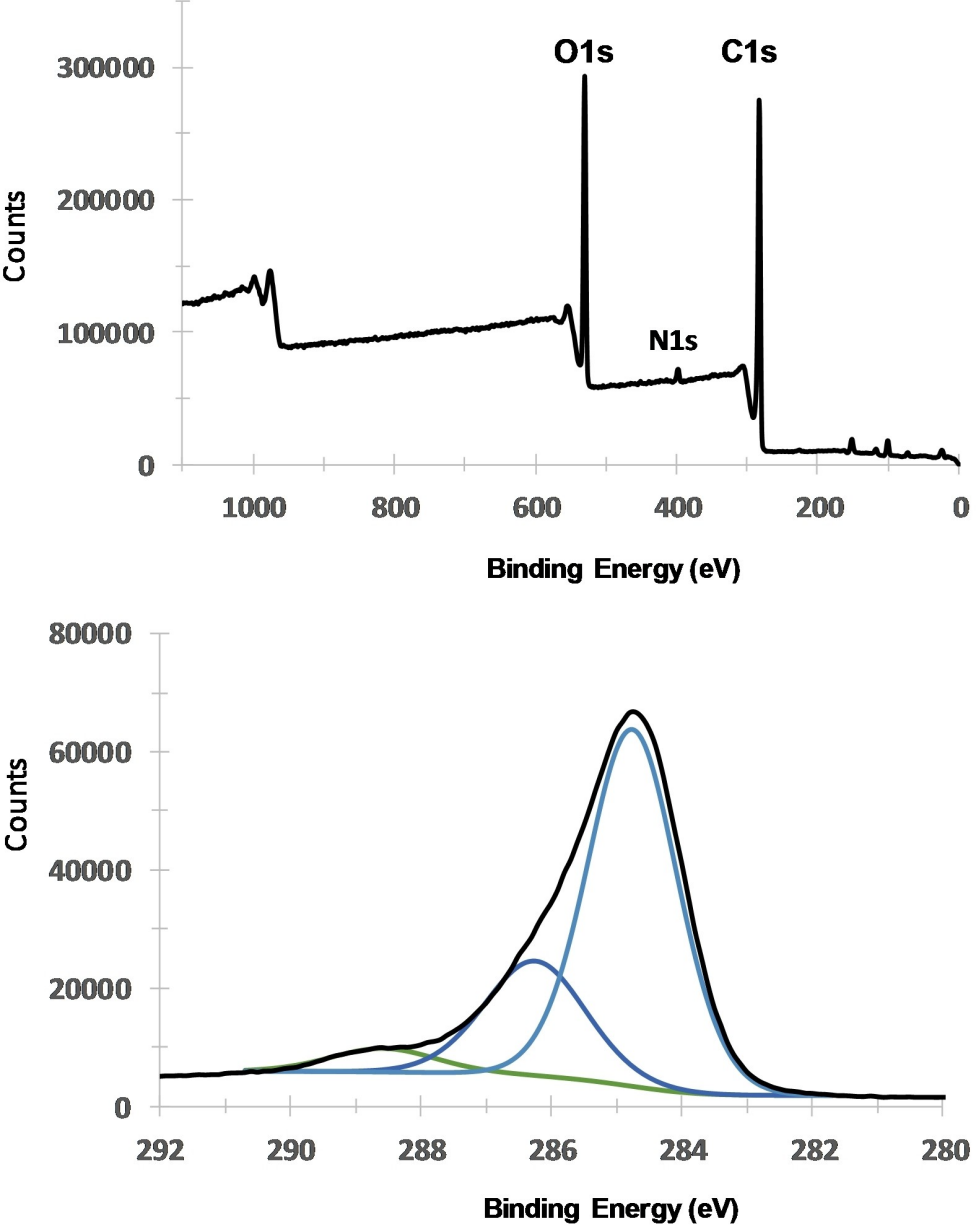
XPS survey and C1s high resolution spectra of bare lignite.

The XPS spectrum of native lignite is recorded before modification, see Figure 1. The XPS survey spectrum presents in addition to C1s (71,0 %), N1s (1.2 %) and O1s (21.8 %) small amounts of Al, (0.9 %) Si, (4.2 %) and Ca (0.7 %). High resolution spectrum of the C1s contribution can be deconvoluted into three peaks 284.7 eV (49.6 %, aromatic and aliphatic carbons), 286.3 eV (18.1 %, C−O) and 288.6 eV (3.8 %, C=O).

### Alkyl layer

**Figure 2:**
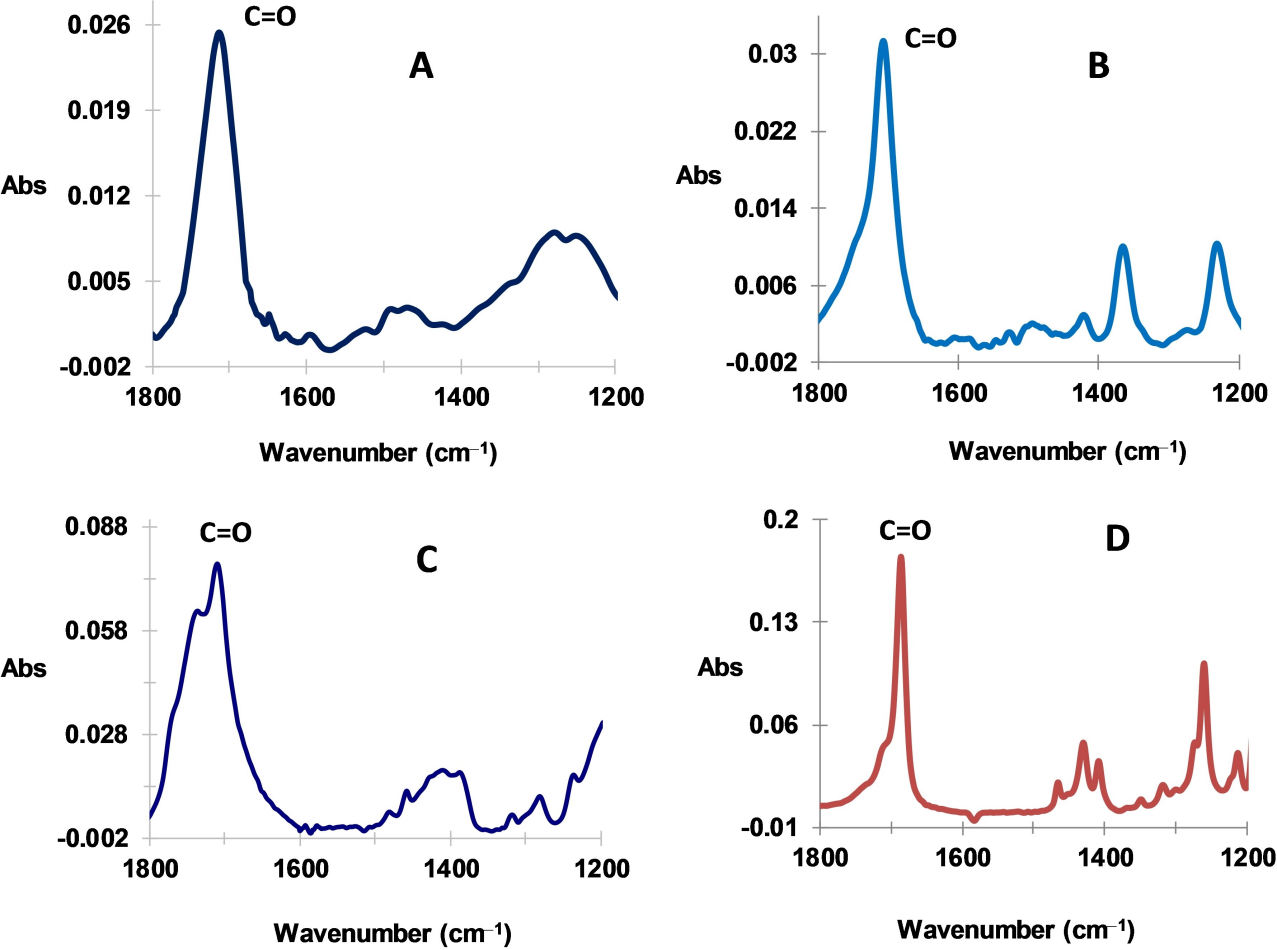
ATR of modified lignites with: A) −C_3_H_6_COOH and B) −C_5_H_10_COOH groups; and ATR of C) IC_3_H_6_COOH and D) BrC_5_H_10_COOH.

Lignite powder is immersed in aqueous acid solution of 0.5 M HCl with 20 mM of 2,6‐dimethylbenzenediazonium chloride (2,6‐DMBD, prepared in situ), or dimethylbenzenediazonium tetrafluoroborate (prepared ex‐situ) and 20 mM of 6‐bromohexanoic acid in the presence of 20 mM solution of potassium iodide (KI)^[31]^ and ultrasonicated during 1 h. The iodine ion reduces the aryldiazonium salts leading to aryl radicals.^[32]^ The modified lignite is rinsed thoroughly in ethanol and acetone and dried at 80 °C. The same procedure is used for the modification with other alkyl halides. Figure 2A and B display the ATR spectra of lignite modified with butanoic and hexanoic groups according to the above procedure. Note that the spectra are recorded with native lignite as a blank, therefore they represent only the grafted film. One observes the presence of strong absorptions band at respectively 1709 and 1705 cm^−1^ attributed to the presence of C=O stretching. By comparison, these bands are observed at respectively, 1706 cm^−1^ and at 1695 cm^−1^ with IC_3_H_8_COOH and BrC_5_H_10_COOH

(Figure 2C and D).^[23]^ Therefore these characteristic bands attest from the presence of alkylcarboxylic groups derived from there corresponding halide.

**Figure 3:**
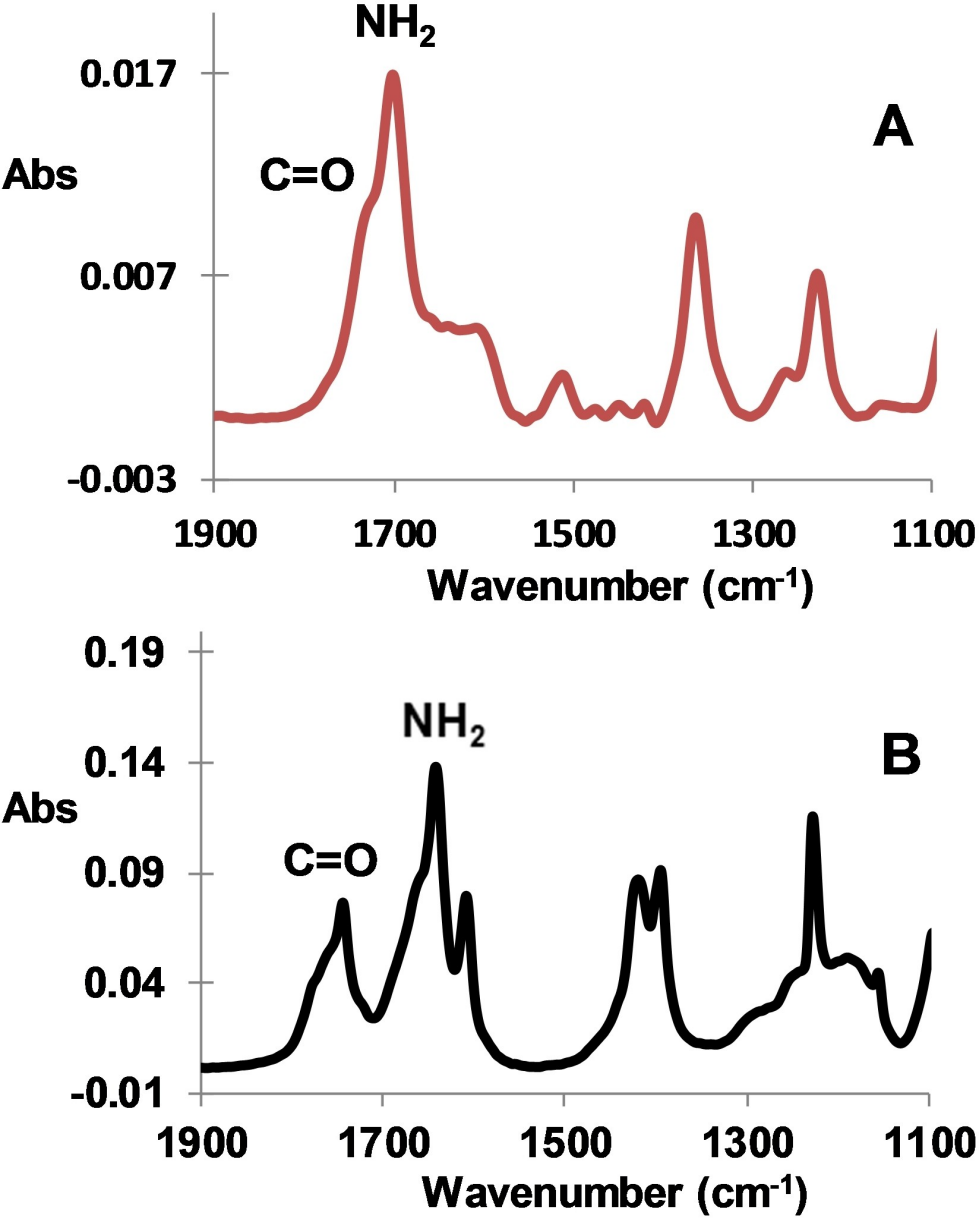
ATR IR spectra of modified lignite with: A) ‐CH_2_CONH_2_ and B) the parent BrCH_2_CONH_2_.

Lignite surface is also modified by the same procedure with acetamide groups issued from 2‐bromoacetamide. Figure 3 shows the ATR spectra of lignite surfaces modified with acetamide groups derived from bromoacetamide along the same procedure as above. One can see the presence of several peaks: 1702 and 1640 cm^−1^ (broad peak) that attest from the presence of C=O and NH_2_ groups when compared with ATR spectrum of BrCH_2_CONH_2_ (respectively at 1740 and 1660 cm^−1^).

**Figure 4:**
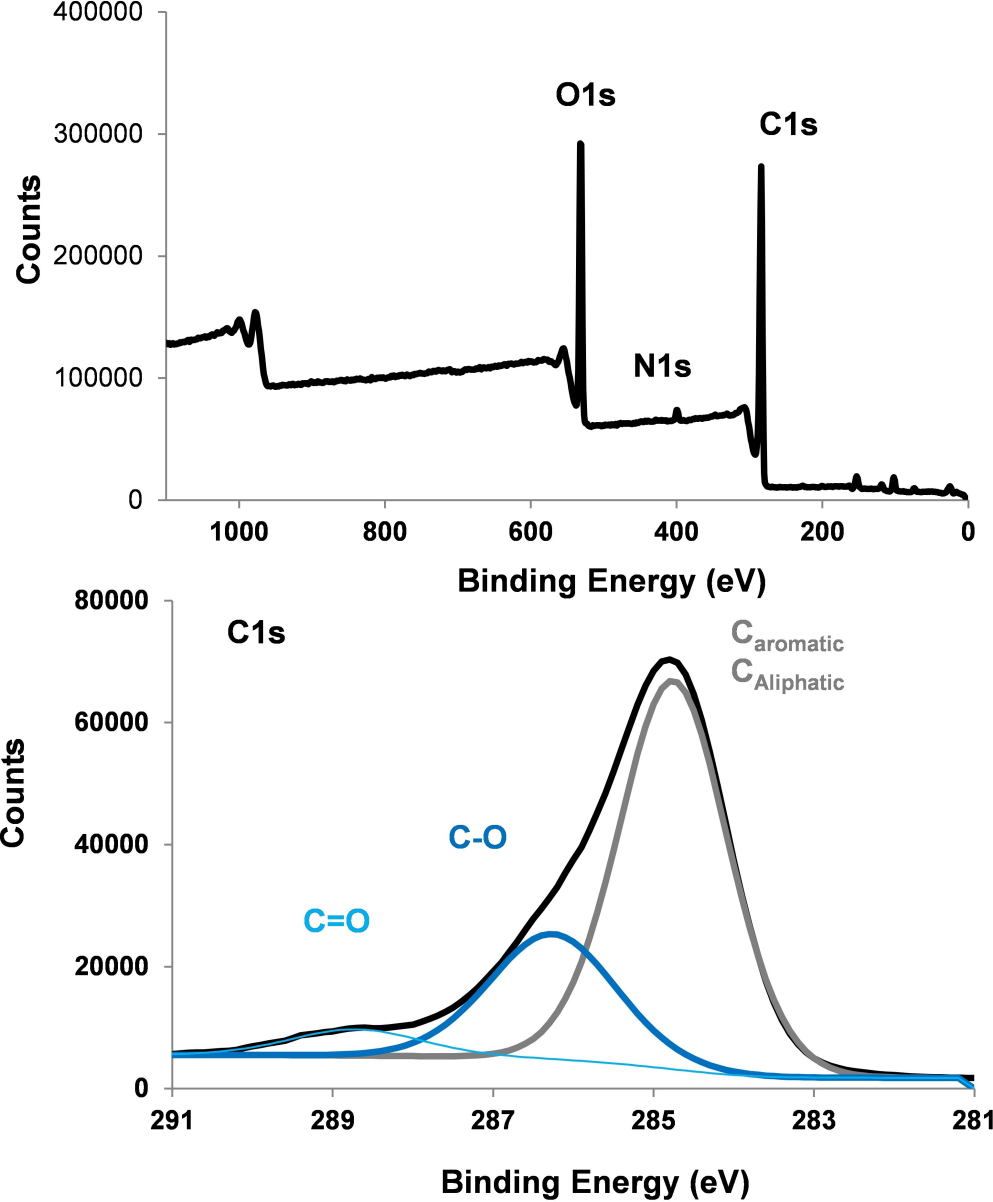
XPS survey and C1s high resolution spectra of lignite modified with hexanoic groups (black curves).

Lignite modified surfaces were also characterized by XPS. The survey spectrum of a –(CH_2_)_5_‐COOH film obtained from 2,6‐DMBD and bromohexanoic acid Br‐(CH_2_)_5_‐COOH indicates the presence of carbon (C1s,71.9 %), nitrogen (N1s, 1.5 %), oxygen (O1s, 25.1 %). The C1s (Figure 4) contribution can be deconvoluted into three peaks 286.3 eV (18.6 %, aromatic and aliphatic carbons), 286.3 eV (18.6 %, C−O) and 288.8 eV (4.0 %, C=O). A similar spectrum is obtained from 2,6‐DMBD and iodobutanoic acid IC_3_H_6_COOH with the same percentage of C=O (4.0 %).^[23]^ When 2,6‐DMBD and bromoacetamide Br‐CH_2_CONH_2_ are reduced on the surface of lignite a similar spectrum is obtained, the C=O contribution at 288.7 eV amounts to 3.9 % and the total N1s contribution to 1.5 %. Note that the presence of bromine Br3d at ~71 eV^[33]^ or iodine I3d_5/2_ at 620 eV are not observed.^[24]^ The absence of halogen atoms in the grafted alkyl layer testifies for the cleavage of the halogen atoms during grafting.

**Figure 5:**
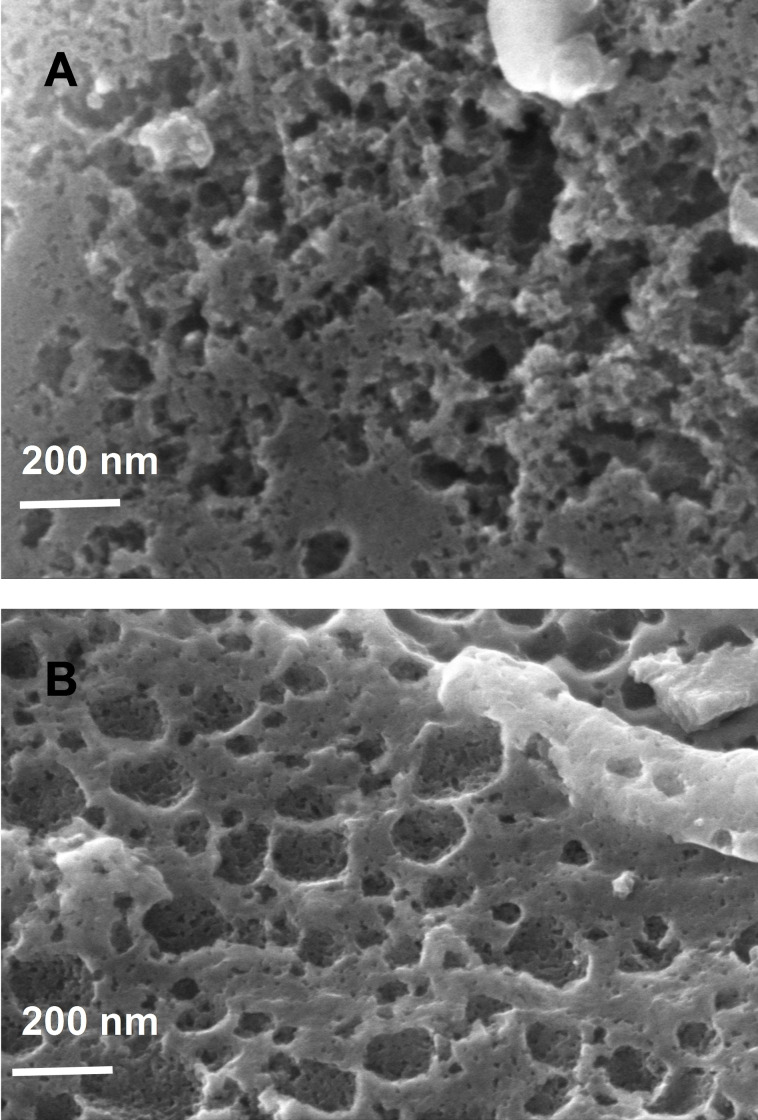
SEM image of lignite A) bare, B) modified with butanoic groups.

The presence of a film on the surface of lignite was also observed by SEM (Scanning Electronic Microscopy), Figure 5 shows a softening of the rough surface of lignite after modification, but the film is thin as it does not fill the porosity.

### Mixed alkyl‐aryl layer

**Figure 6:**
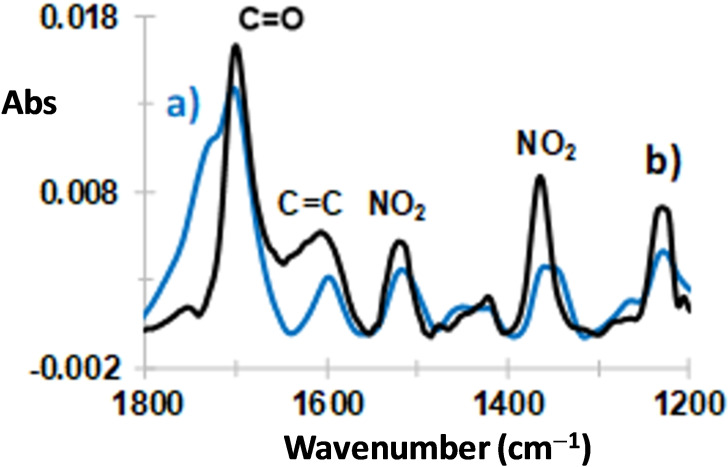
ATR IR spectra of lignite modified with alkyl‐aryl mixed layer containing a) −C_5_H_10_COOH and −C_6_H_4_NO_2_ and b) −C_3_H_8_COOH and −C_6_H_4_NO_2_ groups.

Lignite surface was modified by 6‐bromohexanoic acid and 4‐iodobutanoic in the presence 4‐nitrobenezenediazonium salts (Scheme 1b). ATR IR spectra of these lignite modified samples are shown in Figure 6. One observes the presence of strong absorption bands at respectively 1700 and 1694 cm^−1^, attributed to the presence of C=O stretching. At the difference of Figure 1, several peaks are present at 1600, 1520 and 1360 cm^−1^ assigned respectively to aromatic ring vibrations and asymmetric and symmetric stretching vibrations of NO_2_ group.^[34]^ Therefore these results indicate the formation of a mixed alkyl‐aryl layer.

**Figure 7:**
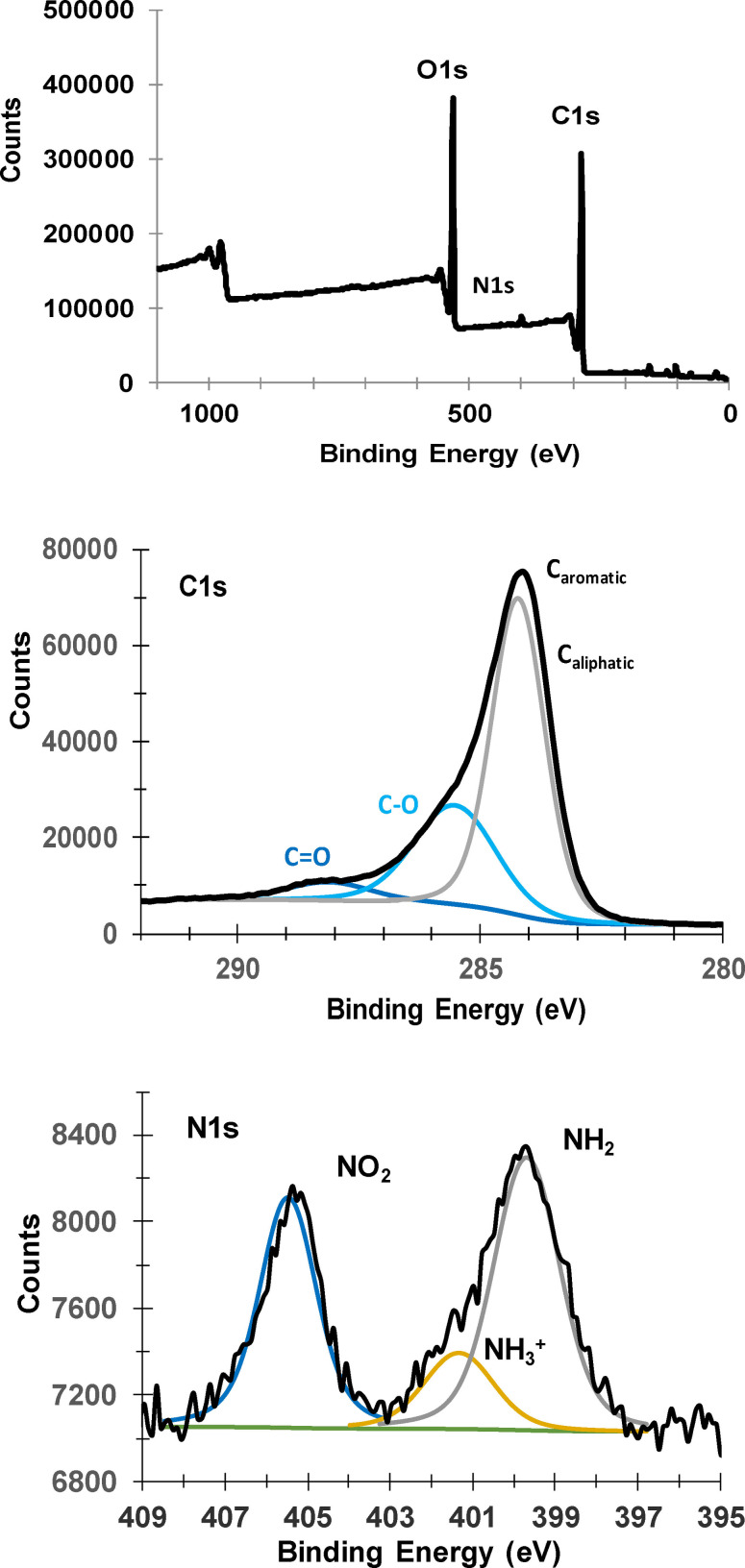
XPS spectra of lignite modified with alkyl‐aryl mixed layer containing −C_5_H_10_COOH and −C_6_H_4_NO_2_. From top to bottom survey spectrum, C1s, N1s.

Lignite, modified with mixed layer containing −C_5_H_10_COOH and −C_6_H_4_NO_2_ groups, is also characterized by XPS (Figure 7). The survey spectrum indicates the presence of carbon (C1s, 69.6 %), nitrogen (N1s, 2.45 %), oxygen (O1s, 26.6 %). The C1s peak can be deconvoluted into three contributions at 284.7 eV (44.9 %, aromatic an aliphatic carbons), 286.2 eV (C−O, 20.3 %) and 288.6 eV (C=O, 4.5 %), the N1s peak can be deconvoluted into three contributions at 399.7 eV (amines, 1.2 %), 401.5 eV (protonated amines, 0.3 %) and 405.5 eV (nitro groups, 0.91 %).^[34]^ These results confirm the presence of carboxylic and nitrophenyl moieties on the surface of lignite.

**Figure 8:**
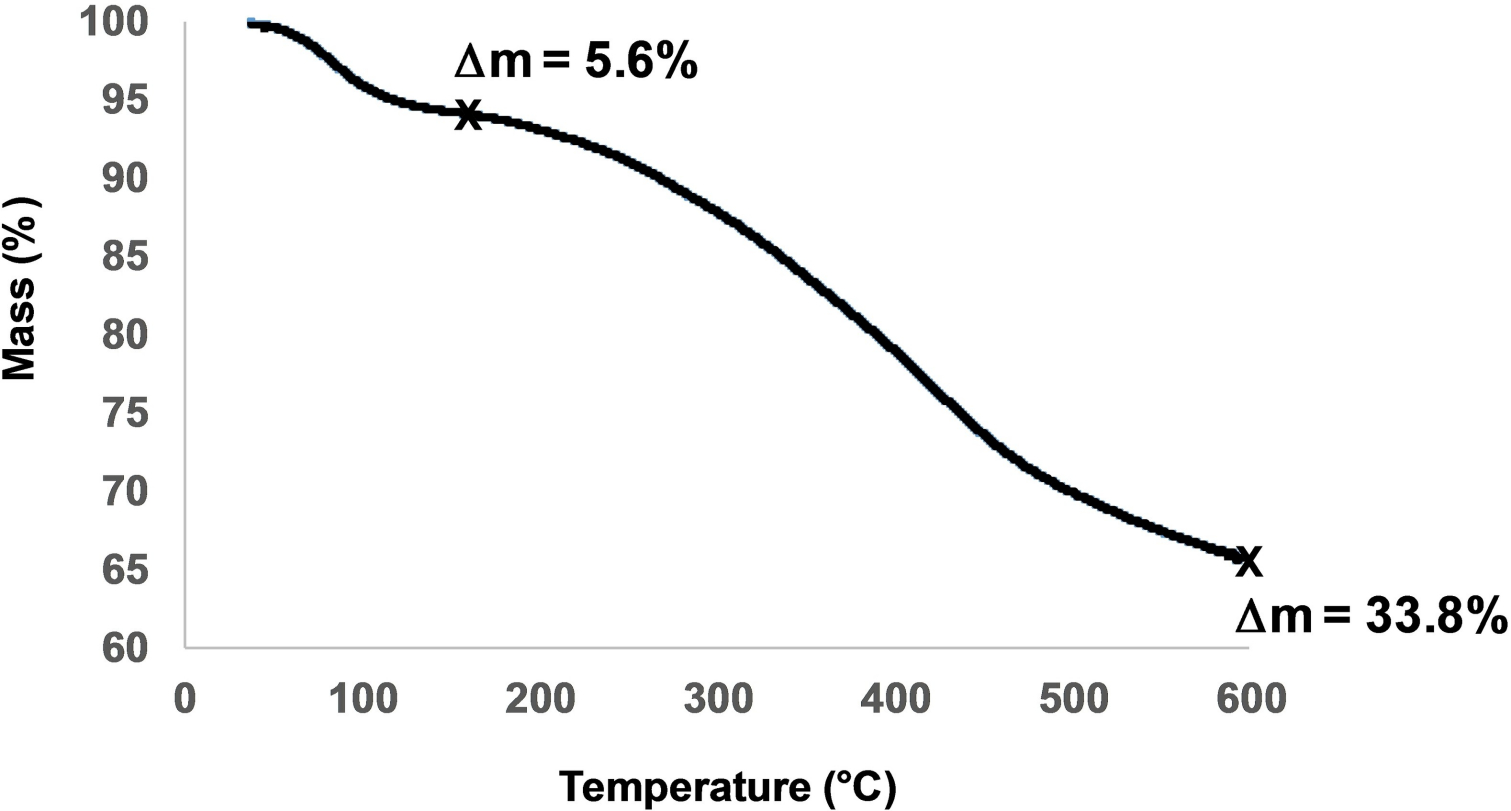
TGA of lignite modified with 4‐nitrobenzenediazonium and bromohexanoic acid.

Lignite modified with 4‐nitrobenzenediazonium and bromohexanoic acid was also analyzed by TGA‐GC‐MS (ThermoGravimetric Analysis‐Gas Chromatography‐Mass Spectroscopy) under He (a very similar TGA is obtained with iodobutyric acid); Figure 8, two mass losses are observed the first one in the 100 °C region corresponds to the loss of adsorbed water, the second one starts at approximately 250 °C and continues up to 600 °C; this second mass loss corresponding to 28.2 %; GC analysis of the gas evolved indicate the presence of CH_4_, CO, CO_2_ (identified by their IR and mass spectra). It is therefore assigned to the thermal cleavage of carboxylic groups, both those from grafted hexanoic groups and that present on the surface of lignite. Indeed, an untreated lignite sample also presents two mass losses the second one starting close to 300 °C and continuing up to 800 °C, it corresponds to 32.1 %; this value is somewhat higher than that of the modified sample indicating that some material is loss during the processing of the sample (washing, dipping in the grafting solution) or that grafting is not very efficient.

**Figure 9:**
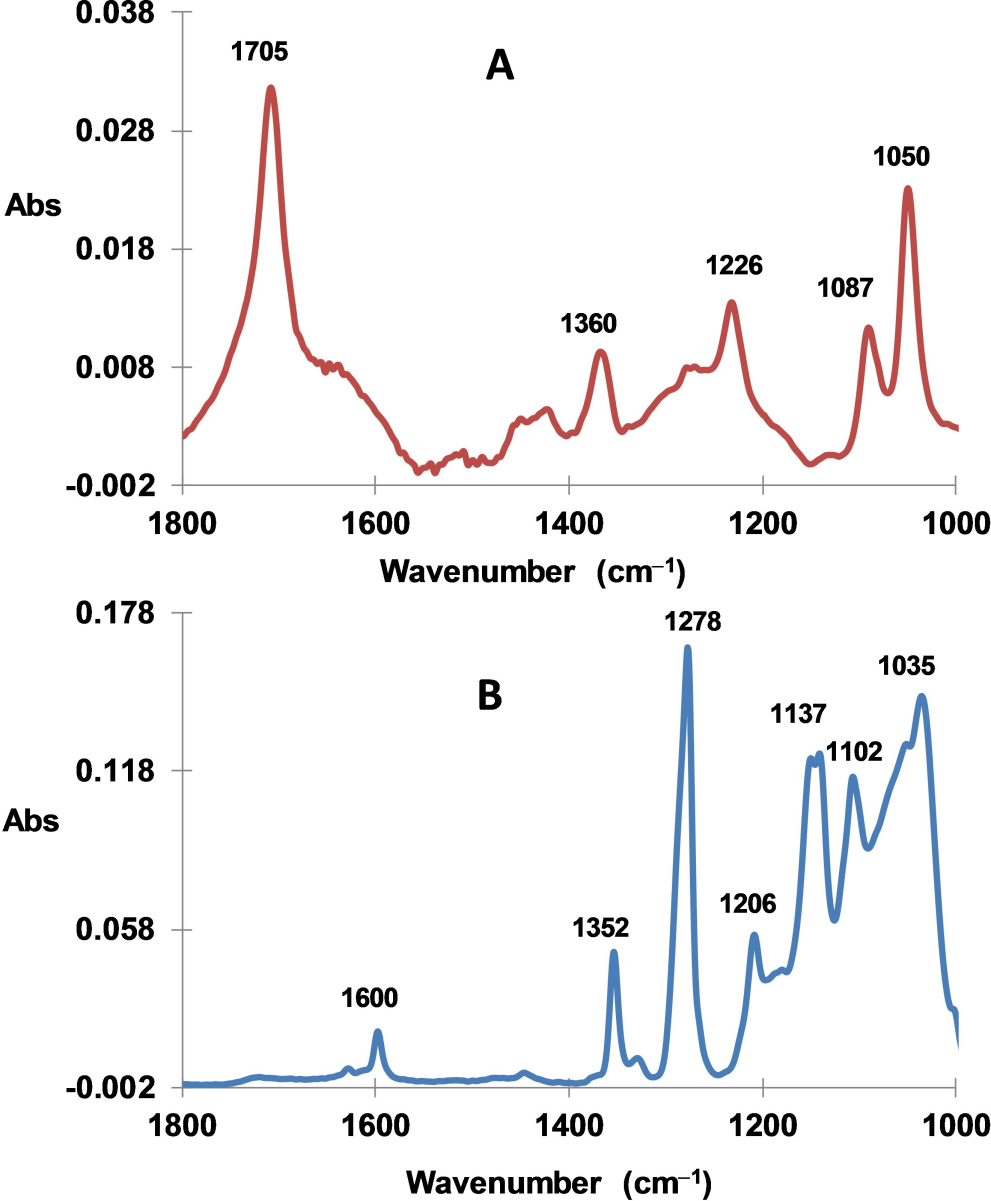
ATR IR spectrum of A) modified lignite with alkyl‐aryl mixed layer containing −C_5_H_10_COOH and 3,5‐(CF_3_)_2_C_6_H_3_‐groups and B) the parent 3,5‐(CF_3_)_2_C_6_H_3_N_2_^+^BF_4_^−^.

Reduction of 3,5‐bis‐trifluoromethylbenzenediazonium salt, 3,5‐(CF_3_)_2_C_6_H_3_N_2_^+^BF_4_^−^ in the presence of bromohexanoic acid Br(CH_2_)_5_COOH was also performed. The IRATR spectrum of such modified lignite is shown in Figure 9A. A peak at 1705 cm^−1^ is attributed to C=O and indicates the presence of hexanoic moieties while other peaks at 1600 (aromatic ring vibration), 1360, 1226, 1087 and 1050 cm^−1^ (CF_3_) are due to the 3,5‐bis‐trifluoromethylphenyl groups (Figure 9B).

**Figure 10:**
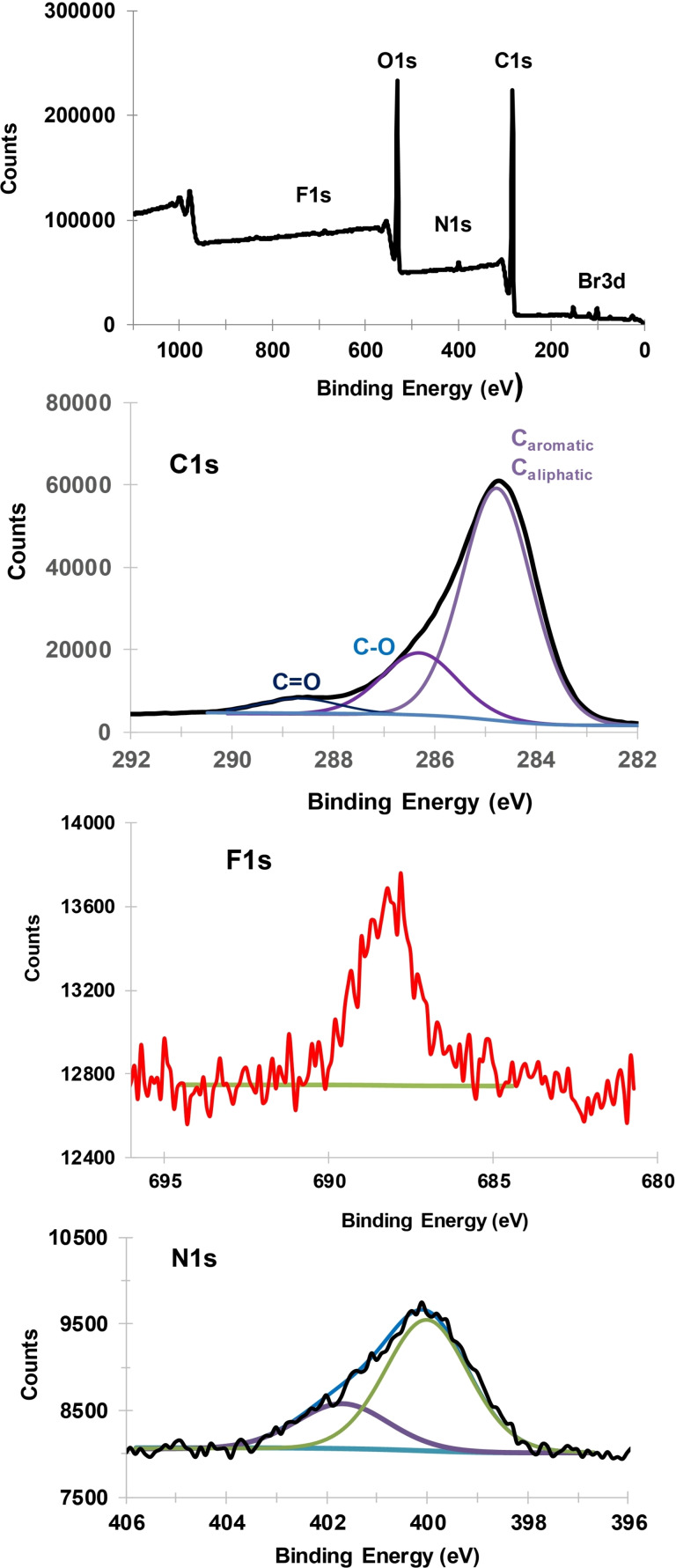
XPS spectra of modified lignite alkyl‐aryl mixed layer containing and −C_5_H_10_COOH and 3,5(CF_3_)_2_C_6_H_3_‐groups. Survey spectrum and high‐resolution spectra of C1s, F1s and N1s.

This modified lignite modified is also characterized by XPS, Figure 10. The survey spectrum indicates the presence of carbon (C1s, 71.9 %), nitrogen (N1s, 1.3 %), oxygen (O1s, 25.2 %), fluorine (F1s, 0.3 %). The C1s peak can be deconvoluted into three contributions at 284.8 eV (52.5 %, aromatic an aliphatic carbons), 286.3 eV (C−O, 15.3 %) and 288.7 eV (C=O, 4.1 %), the N1s peak can be deconvoluted into two contributions at 400.0 eV (amines, 1.0 %), 401.7 eV (protonated amines, 0.4 %).^[34]^ Note as before the low surface concentration of the 3,5‐(CF_3_)_2_C_6_H_3_‐groups.

Modification of the surface lignite with alkyl or alkyl‐aryl layers is attested by XPS, ATR spectroscopic measurements; the characteristic data are gathered in Table 1.

**Table 1:** IRATR and XPS data supporting the modification of lignite.^[a]^

	IR [cm^−1^]	XPS		
		C1s (C=O) [%]	N1s [400+402 eV]	Other
Blank	1741	3.8	1.2	
Modified Lignite Surface	[b]		N1s [406 eV]	F1s [688 eV]
−(CH_2_)_3_COOH	1709 (C=O)	4.0	–	
−(CH_2_)_5_COOH	1705 (C=O)	4.4	–	
−CH_2_CONH_2_	1702 (C=O, 1640 (NH_2_)	3.9	1.5	
‐nitrophenyl+−(CH_2_)_5_COOH	1520,1360 (NO_2_), 1700 (C=O)	4.5	0.9	
‐3,5‐bis trifluorophenyl+ −(CH_2_)_5_COOH	1520,1360 (NO_2_), 11694 (C=O)	4.1		0.3

[a] The only characteristic data of the modifying layer are reported, [b] the IR ATR spectra are recorded with unmodified lignite as a blank, therefore the C=O bands only pertain to the modifying film.

### DFT calculations

**Figure 11:**
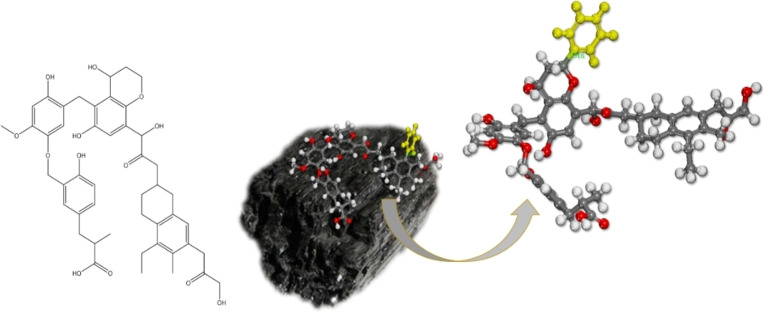
Selected lignite molecular model used for BDE calculations.

It has been demonstrated that the computation of the Bond Dissociation Energy, or BDE, is useful for determining how stable is the newly created interface.[^35^, ^36^] To compute the BDE, a simple model of the surface lignite (presented in Figure 11) is grafted covalently by a phenyl group. The model of Kosovo lignite is the same as it was used previously.^[37]^

**Figure 12:**
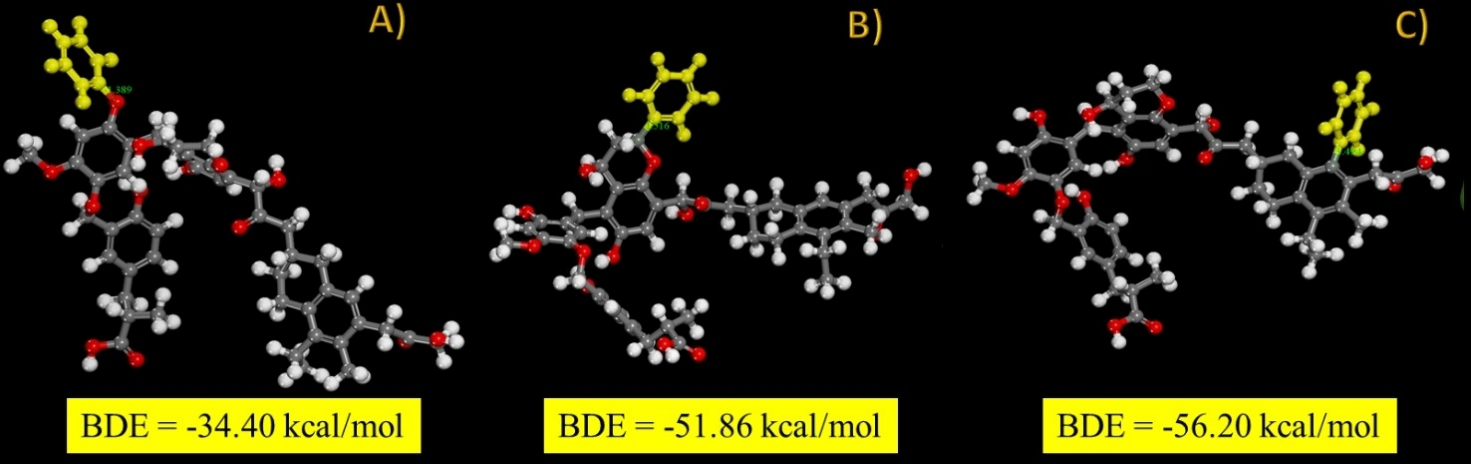
The assessment of BDE for the grafting of phenyl radical on three unique grafting sites onto the liginit model with corresponding BDE values.

**Figure 13:**
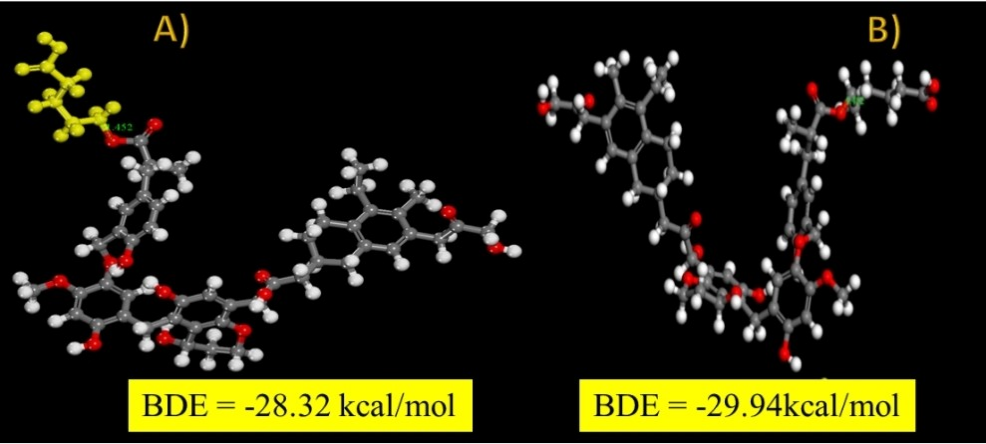
The assessment of BDE for the grafting of: A) pentanoic an B) hexanoic radicals on two unique grafting sites onto the liginite model with corresponding BDE values.

We performed the modeling in three different cases: either aryl radicals attack the surface OH groups of lignite and generate C−O bonds or they attack a carbon of an aryl or heterocyclic cycle; Figure 12 presents these three possible modifications. The optimum geometrical structures of modified lignite, are presented in Figure 13. The equation^[1]^ is used to determine the BDE between the aryl group and the lignite surface:[^27^, ^31^]

(1) $$BDE=\left[E_{Lignite-O\;or\;C-Ph}+E_{H}\right]-\left[E_{Lignite}+E_{Ph^{*}}\right]$$

where *E*_*lignite‐O or C‐Ph*_ denotes BDE for the grafting of, respectively, a phenyl group on an oxygen or carbon atom of the model; *E_H_* ‐ BDE of H in C_6_H_6_, *E_liginte_* ‐ optimized lignite energy and *E*_Ph*_ ‐ phenyl radical energy. The BDE value in this case is ‐56.20 kcal/mol, a value that signpost to much stronger interface than that formed by aryl radicals in the case of other surfaces such as: gold cluster (BDE_Au‐Ph_=−34.4 kcal/mol)^[27]^ and carbon (in the literature).

Similarly, BDE is assessed in the grafting of pentanoic and hexanoic radicals on lignite. For the formation of an O_lignite_‐C_hexanoic_ (Figure 13A) and C_lignite_‐C_hexanoic_ (Figure 13B), the attachment of alkyl radicals results in a somewhat less stable interface than with aryl radicals.

The BDE values for pentanoic and hexanoic are −28.32 and −29.94 kcal/mol, respectively. The resulting values are lower than the value obtained during the binding an aryl radical (47.18 kcal/mol). This finding is significant because it demonstrates that a value of BDE of this magnitude is adequate for the formation of stable organic layers bonded to the surface of lignite; it is larger than the bond strength of aryl groups attached to the surface of various metals.^[38]^

We also calculated the BDE of alkyl radicals bonded to a carbon atom of the lignite structure: C_lignite_‐C_alkyl_ 37.21 kcal/mol. This value is lower than that obtained during the surface binding of an aryl radical (46.37 kcal/mol), but larger than the previous value of O_lignite_‐C_alkyl_ interface. Based on the calculations, we can conclude that, even in the case of the attachment of the alkyl group (pentanoic or hexanoic radical) to the surface of the lignite, it is more likely that the modification occurs on the carbon atoms of the structural part, rather than on the functional groups present in lignite. This is significant because, after modification, such functional groups stay intact, and contribute to the reactivity and hydrophilic qualities of the modified lignite surface.^[38]^ As a result of the reaction of 6‐bromo‐1‐hexanoic acid with radicals generated by the 2,6‐dimethylbenzenediazonium salt on the lignite surface coated with alkyl carboxylic groups, a nanocomposite material is created that will be tested for the creation of asymmetric heterogeneous membranes.

## Conclusions

Tethering of the surface lignite with alkyl or alkyl‐aryl layers is possible by the use of aryl diazonium salts as source of aryl radicals. Aryl diazonium salts without steric hindrance such as 4‐nitrobenzenediazonium salt and 3,5‐bis‐trifluoromethylbenzene diazonium in the presence of bromohexanoic acid permit to modify the lignite surface with mixed alkyl‐aryl layer. With the 2,6‐DMBD as the diazonium salt the only pentanoic or hexanoic groups are attached to the surface of lignite. The formation of a stable interface (critical for practical applications) during the grafting reactions of alkyl and aryl moieties with the lignite surface has been confirmed by the calculated BDE. Lignite is a highly oxidized material (21.8 % oxygen on the surface as measured by XPS) therefore, as indicated by the DFT calculations grafting can take place either on oxygen or carbon the two major components of the surface. The grafting on minor components (Al, Si) cannot be excluded. It should be also noted that the surface modification of lignite is difficult, for example the C=O group of alkylcarboxylic acid are clearly evidenced by IRATR, but the C=O% measured by XPS is close to that of bare lignite; the amount of aromatic rings (nitro or trifluorophenyl) is more significant. These surface modification of lignite can be applied to membranes or trapping of metallic pollution.

## Conflict of interests

The authors declare no conflict of interest.

1

## Supporting information

As a service to our authors and readers, this journal provides supporting information supplied by the authors. Such materials are peer reviewed and may be re‐organized for online delivery, but are not copy‐edited or typeset. Technical support issues arising from supporting information (other than missing files) should be addressed to the authors.

Supporting Information

## Data Availability

The data that support the findings of this study are available from the corresponding author upon reasonable request.
