# Supplementary material for: Surface Modification of Lignite with Alkyl and Mixed Alkyl‐Aryl Films Generated from an Aryl Diazonium Salt and Alkyl Halides: Experimental Results and Theoretical Analyses
Source: ChemistryOpen. 2023 Dec 5;13(4):e202300134. doi: 10.1002/open.202300134 (PMC11004459; doi:10.1002/open.202300134)
Supplement: Supplementary file 1 — Supporting Information [file OPEN-13-e202300134-s001.pdf]

ChemistryOpen

Supporting Information

Surface Modification of Lignite with Alkyl and Mixed Alkyl-Aryl Films Generated from an Aryl Diazonium Salt and Alkyl Halides: Experimental Results and Theoretical Analyses

Msc. Gentiana Hasani, Avni Berisha, Dardan Hetemi., Philippe Decorse, Jean Pinson, and Fetah I. Podvorica*

Structure (Figure 12 A)

E = -3030.0388596 Ha

O1	20.8569000000	-31.2972000000	-0.4056610000
O2	19.3099000000	-30.0972000000	0.7365330000
O3	22.3327000000	-22.8154000000	-2.1958000000
O4	37.9328000000	-32.1186000000	0.5846870000
O5	35.0525000000	-32.3605000000	-1.5061400000
O6	28.9152000000	-24.8497000000	0.9338880000
O7	30.0591000000	-23.1721000000	-1.9392500000
O8	25.3122000000	-19.6444000000	2.1962300000
O9	29.0363000000	-21.3561000000	1.4936700000
O10	21.2775000000	-22.7295000000	0.9405360000
O11	25.2600000000	-22.0113000000	-2.5128800000
O12	22.9625000000	-17.7748000000	-0.8831440000
O13	19.4742000000	-20.6669000000	0.9117070000
C14	22.2020000000	-15.5809000000	-1.3582600000
C15	21.7645000000	-14.3245000000	-0.9366770000
C16	21.5906000000	-14.0566000000	0.4240270000
C17	21.8691000000	-15.0520000000	1.3662900000
C18	22.3174000000	-16.3105000000	0.9611160000
C19	22.4682000000	-16.5675000000	-0.4061080000
C20	22.6959000000	-29.0619000000	-0.2465730000
C21	20.4847000000	-30.2271000000	0.0400498000
C22	21.1832000000	-28.8912000000	-0.1236290000
C23	20.5624000000	-28.1765000000	-1.3635700000
C24	21.9036000000	-24.0938000000	-1.9050000000
C25	22.4054000000	-25.1111000000	-2.7215200000
C26	21.9824000000	-26.4275000000	-2.5478500000

C27	21.0550000000	-26.7634000000	-1.5514600000
C28	20.5891000000	-25.7327000000	-0.7280290000
C29	20.9839000000	-24.3972000000	-0.8766580000
C30	37.2413000000	-32.4867000000	-0.6092580000
C31	35.7777000000	-32.0464000000	-0.5753780000
C32	35.3328000000	-31.2252000000	0.6240680000
C33	33.0507000000	-33.0335000000	1.1613400000
C34	29.8204000000	-32.7138000000	-0.1340490000
C35	30.2971000000	-31.9106000000	1.0869700000
C36	33.6852000000	-29.3995000000	0.2616080000
C37	33.9107000000	-30.7353000000	0.5841320000
C38	32.8079000000	-31.5763000000	0.8541360000
C39	31.5024000000	-31.0378000000	0.7941400000
C40	32.2561000000	-27.4018000000	-0.2198080000
C41	32.4019000000	-28.8567000000	0.1726730000
C42	31.2959000000	-29.6825000000	0.4322460000
C43	29.8919000000	-29.1112000000	0.3436810000
C44	29.8040000000	-27.7715000000	-0.3900330000
C45	30.8825000000	-26.7988000000	0.0962925000
C46	30.7556000000	-25.4135000000	-0.5342980000
C47	29.6560000000	-24.5287000000	0.0189144000
C48	29.5964000000	-23.1048000000	-0.5792100000
C49	26.6522000000	-19.6595000000	1.6829300000
C50	27.6609000000	-19.8632000000	2.8172100000
C51	29.0370000000	-20.1310000000	2.2493400000
C52	20.3435000000	-23.3574000000	0.0067165000
C53	26.8649000000	-20.6819000000	0.5717780000
C54	28.0308000000	-21.4728000000	0.5581300000

C55	28.2581000000	-22.4137000000	-0.4631740000
C56	27.2702000000	-22.6067000000	-1.4241900000
C57	26.1119000000	-21.8271000000	-1.4400800000
C58	25.9296000000	-20.8059000000	-0.4863230000
C59	24.8766000000	-19.7492000000	-0.7202240000
C60	18.5221000000	-19.6014000000	0.8549060000
C61	22.5266000000	-18.9650000000	-0.3175340000
C62	23.4688000000	-19.9979000000	-0.2341290000
C63	23.0131000000	-21.2360000000	0.2319640000
C64	21.6744000000	-21.4628000000	0.5639010000
C65	20.7534000000	-20.3866000000	0.5167790000
C66	21.1902000000	-19.1432000000	0.0571048000
H67	22.3430000000	-15.8173000000	-2.4126100000
H68	21.5567000000	-13.5512000000	-1.6771500000
H69	21.2449000000	-13.0748000000	0.7499780000
H70	21.7510000000	-14.8430000000	2.4303200000
H71	22.5572000000	-17.0868000000	1.6881400000
H72	18.9143000000	-30.9930000000	0.7428950000
H73	23.1775000000	-28.0837000000	-0.3745150000
H74	23.1167000000	-29.5373000000	0.6495220000
H75	22.9463000000	-29.6935000000	-1.1098300000
H76	20.9412000000	-28.2852000000	0.7636020000
H77	19.4668000000	-28.1703000000	-1.2527900000
H78	20.7899000000	-28.7754000000	-2.2593800000
H79	22.0309000000	-22.1909000000	-1.5038500000
H80	23.1192000000	-24.8437000000	-3.5012600000
H81	22.3758000000	-27.2071000000	-3.2029100000
H82	19.8669000000	-25.9646000000	0.0596069000

H83	38.8583000000	-32.3931000000	0.4907790000
H84	37.2421000000	-33.5789000000	-0.7726270000
H85	37.6889000000	-32.0208000000	-1.5066800000
H86	36.0338000000	-30.3777000000	0.7042720000
H87	35.5562000000	-31.8275000000	1.5222800000
H88	33.9499000000	-33.1769000000	1.7776300000
H89	32.2161000000	-33.5009000000	1.6968100000
H90	33.2053000000	-33.6053000000	0.2318840000
H91	30.5979000000	-33.4055000000	-0.4874750000
H92	28.9228000000	-33.3045000000	0.1035390000
H93	29.5756000000	-32.0460000000	-0.9727840000
H94	30.5240000000	-32.6018000000	1.9116900000
H95	29.4707000000	-31.2880000000	1.4548400000
H96	34.5448000000	-28.7531000000	0.0621519000
H97	33.0506000000	-26.8139000000	0.2686030000
H98	32.4406000000	-27.3106000000	-1.3072100000
H99	29.4875000000	-28.9830000000	1.3642300000
H100	29.2233000000	-29.8313000000	-0.1500320000
H101	28.8037000000	-27.3396000000	-0.2455780000
H102	29.9349000000	-27.9304000000	-1.4759700000
H103	30.7803000000	-26.6941000000	1.1905100000
H104	30.6233000000	-25.4793000000	-1.6276500000
H105	31.6970000000	-24.8433000000	-0.4190290000
H106	29.9999000000	-22.2705000000	-2.2976700000
H107	30.3166000000	-22.5330000000	0.0388666000
H108	25.0519000000	-20.5682000000	2.3599900000
H109	26.7863000000	-18.6445000000	1.2698200000
H110	27.3528000000	-20.7269000000	3.4273700000

H111	27.6651000000	-18.9822000000	3.4739800000
H112	29.7896000000	-20.2776000000	3.0334300000
H113	29.3703000000	-19.3054000000	1.5944100000
H114	19.5717000000	-23.8330000000	0.6239140000
H115	19.8515000000	-22.5732000000	-0.5880490000
H116	24.3282000000	-22.1088000000	-2.2308000000
H117	27.4181000000	-23.3331000000	-2.2229800000
H118	24.8271000000	-19.5856000000	-1.8108000000
H119	25.2137000000	-18.7946000000	-0.2955490000
H120	18.8270000000	-18.7575000000	1.4927200000
H121	18.3748000000	-19.2499000000	-0.1784620000
H122	17.5856000000	-20.0255000000	1.2319200000
H123	23.7037000000	-22.0782000000	0.3196290000
H124	20.5057000000	-18.3011000000	-0.0244470000

Structure (Figure 12 B)

E = -3030.0424553 Ha

O1	20.6737000000	-31.0485000000	-0.4147630000
O2	19.1413000000	-29.7660000000	0.6525830000
O3	22.3570000000	-22.5320000000	-2.0594100000
O4	38.0055000000	-32.2004000000	0.8836600000
O5	35.3474000000	-32.1941000000	-1.4954700000
O6	28.9075000000	-25.0053000000	0.8841190000
O7	30.1570000000	-23.3541000000	-1.9380700000
O8	25.6659000000	-19.5473000000	2.2190700000
O9	29.2323000000	-21.5008000000	1.5018200000
O10	21.5237000000	-22.5791000000	1.1784600000
O11	25.3816000000	-22.0195000000	-2.4466000000

O12	23.3406000000	-17.6004000000	-0.4567420000
O13	19.8552000000	-20.4259000000	1.4365600000
C14	30.4316000000	-19.8974000000	4.5269500000
C15	31.4798000000	-20.0992000000	5.4306700000
C16	32.5789000000	-20.8787000000	5.0709400000
C17	32.6265000000	-21.4520000000	3.7963000000
C18	31.5836000000	-21.2439000000	2.8937100000
C19	30.4686000000	-20.4692000000	3.2482300000
C20	22.5723000000	-28.8817000000	-0.3996910000
C21	20.3286000000	-29.9543000000	-0.0090451800
C22	21.0806000000	-28.6480000000	-0.1783780000
C23	20.4211000000	-27.8535000000	-1.3462500000
C24	21.8966000000	-23.8045000000	-1.7944100000
C25	22.2938000000	-24.8003000000	-2.6918600000
C26	21.8291000000	-26.1060000000	-2.5484500000
C27	20.9644000000	-26.4550000000	-1.5021900000
C28	20.6065000000	-25.4486000000	-0.5980440000
C29	21.0457000000	-24.1238000000	-0.7128540000
C30	37.4201000000	-32.4700000000	-0.3903270000
C31	35.9592000000	-32.0250000000	-0.4527330000
C32	35.3690000000	-31.3949000000	0.7998850000
C33	33.0914000000	-33.2402000000	1.0307900000
C34	29.8946000000	-32.7909000000	-0.5242060000
C35	30.3336000000	-32.1460000000	0.8018330000
C36	33.7396000000	-29.5519000000	0.4143020000
C37	33.9535000000	-30.9036000000	0.6656300000
C38	32.8454000000	-31.7713000000	0.7770740000
C39	31.5429000000	-31.2412000000	0.6577980000

C40	32.3264000000	-27.5447000000	-0.0684403000
C41	32.4595000000	-29.0157000000	0.2589290000
C42	31.3473000000	-29.8649000000	0.3806240000
C43	29.9478000000	-29.2933000000	0.2458790000
C44	29.8940000000	-27.9270000000	-0.4413730000
C45	30.9252000000	-26.9625000000	0.1513850000
C46	30.8395000000	-25.5672000000	-0.4643750000
C47	29.7103000000	-24.6836000000	0.0245563000
C48	29.7057000000	-23.2578000000	-0.5754970000
C49	26.9854000000	-19.6498000000	1.6654800000
C50	28.0039000000	-19.8748000000	2.7896500000
C51	29.3636000000	-20.2541000000	2.2328300000
C52	20.5110000000	-23.1110000000	0.2679730000
C53	27.1097000000	-20.7019000000	0.5752660000
C54	28.2291000000	-21.5517000000	0.5611080000
C55	28.4026000000	-22.5067000000	-0.4557350000
C56	27.3937000000	-22.6671000000	-1.4011000000
C57	26.2695000000	-21.8378000000	-1.4056100000
C58	26.1519000000	-20.7943000000	-0.4647560000
C59	25.1217000000	-19.7098000000	-0.6710760000
C60	18.9705000000	-19.3100000000	1.5408700000
C61	22.8725000000	-18.8090000000	0.0025788700
C62	23.7499000000	-19.9020000000	-0.0676183000
C63	23.2725000000	-21.1367000000	0.3826380000
C64	21.9620000000	-21.3146000000	0.8379980000
C65	21.1085000000	-20.1916000000	0.9405120000
C66	21.5779000000	-18.9446000000	0.5164680000
H67	29.5800000000	-19.2926000000	4.8375700000

H68	31.4311000000	-19.6473000000	6.4225800000
H69	33.3935000000	-21.0426000000	5.7773000000
H70	33.4783000000	-22.0682000000	3.5043700000
H71	31.6195000000	-21.7034000000	1.9066600000
H72	18.7157000000	-30.6473000000	0.6738930000
H73	23.0910000000	-27.9206000000	-0.5131100000
H74	23.0216000000	-29.4191000000	0.4462360000
H75	22.7429000000	-29.4811000000	-1.3051000000
H76	20.9213000000	-28.0668000000	0.7447700000
H77	19.3354000000	-27.8084000000	-1.1715200000
H78	20.5721000000	-28.4182000000	-2.2801300000
H79	22.1574000000	-21.9365000000	-1.3073100000
H80	22.9620000000	-24.5236000000	-3.5078100000
H81	22.1423000000	-26.8675000000	-3.2659600000
H82	19.9325000000	-25.6914000000	0.2283960000
H83	38.9372000000	-32.4675000000	0.8451280000
H84	37.4287000000	-33.5485000000	-0.6318610000
H85	37.9443000000	-31.9472000000	-1.2108600000
H86	36.0434000000	-30.5702000000	1.0854900000
H87	35.4945000000	-32.1246000000	1.6180000000
H88	33.4033000000	-33.4254000000	2.0724400000
H89	32.2049000000	-33.8552000000	0.8461320000
H90	33.8831000000	-33.6252000000	0.3740730000
H91	30.7089000000	-33.3812000000	-0.9678930000
H92	29.0301000000	-33.4541000000	-0.3757980000
H93	29.6126000000	-32.0276000000	-1.2639900000
H94	30.5434000000	-32.9339000000	1.5387600000
H95	29.4942000000	-31.5749000000	1.2239000000

H96	34.6043000000	-28.8868000000	0.3279600000
H97	33.0668000000	-26.9735000000	0.5161430000
H98	32.6064000000	-27.3985000000	-1.1286800000
H99	29.4954000000	-29.2044000000	1.2509700000
H100	29.3043000000	-29.9923000000	-0.3071100000
H101	28.8810000000	-27.5091000000	-0.3509730000
H102	30.0999000000	-28.0469000000	-1.5203000000
H103	30.7334000000	-26.8798000000	1.2348800000
H104	30.7723000000	-25.6220000000	-1.5641500000
H105	31.7736000000	-25.0004000000	-0.2870970000
H106	30.1282000000	-22.4540000000	-2.3039900000
H107	30.4558000000	-22.7168000000	0.0339039000
H108	25.3538000000	-20.4517000000	2.4011500000
H109	27.1607000000	-18.6529000000	1.2235300000
H110	27.6528000000	-20.6927000000	3.4379300000
H111	28.0501000000	-18.9624000000	3.3974500000
H112	29.6962000000	-19.4855000000	1.5054800000
H113	19.7567000000	-23.5914000000	0.9030920000
H114	20.0190000000	-22.2699000000	-0.2464940000
H115	24.4521000000	-21.9384000000	-2.1552800000
H116	27.4945000000	-23.4147000000	-2.1874800000
H117	25.0004000000	-19.5744000000	-1.7612500000
H118	25.5211000000	-18.7551000000	-0.3029370000
H119	22.6561000000	-16.9264000000	-0.3142250000
H120	19.3620000000	-18.5521000000	2.2389600000
H121	18.7857000000	-18.8498000000	0.5570490000
H122	18.0312000000	-19.7127000000	1.9347000000
H123	23.9231000000	-22.0140000000	0.3620860000

H124	20.9358000000	-18.0650000000	0.5753620000

Structure (Figure 12 C)

E = -3030.0494565 Ha

O1	20.7807000000	-30.9988000000	-0.4536910000
O2	19.2667000000	-29.7969000000	0.7294500000
O3	22.2283000000	-22.4651000000	-2.0765200000
O4	37.8142000000	-32.5595000000	1.3234200000
O5	35.5109000000	-32.1185000000	-1.3597400000
O6	28.8295000000	-25.0943000000	0.7543880000
O7	30.0590000000	-23.3864000000	-2.0263100000
O8	25.6444000000	-19.6063000000	2.2381100000
O9	29.2270000000	-21.5244000000	1.4250200000
O10	21.4221000000	-22.5563000000	1.1825500000
O11	25.2799000000	-22.0473000000	-2.4327000000
O12	23.3145000000	-17.6041000000	-0.4498350000
O13	19.7777000000	-20.3810000000	1.4151700000
C14	35.4535000000	-27.8897000000	1.2825400000
C15	36.5753000000	-27.0776000000	1.0952500000
C16	37.2124000000	-27.0353000000	-0.1484870000
C17	36.7233000000	-27.8139000000	-1.2014200000
C18	35.5996000000	-28.6240000000	-1.0146500000
C19	34.9501000000	-28.6733000000	0.2297210000
C20	22.6226000000	-28.7761000000	-0.3972540000
C21	20.4232000000	-29.9282000000	0.0023196200
C22	21.1234000000	-28.5943000000	-0.1711510000
C23	20.4313000000	-27.8313000000	-1.3413800000

C24	21.7962000000	-23.7470000000	-1.8088400000
C25	22.2124000000	-24.7336000000	-2.7073700000
C26	21.7804000000	-26.0499000000	-2.5591900000
C27	20.9302000000	-26.4176000000	-1.5074200000
C28	20.5496000000	-25.4183000000	-0.6050330000
C29	20.9579000000	-24.0837000000	-0.7233040000
C30	37.4427000000	-32.5451000000	-0.0549580000
C31	36.0158000000	-32.0355000000	-0.2498330000
C32	35.3319000000	-31.4321000000	0.9637150000
C33	33.0642000000	-33.2301000000	1.1196000000
C34	29.9487000000	-32.8471000000	-0.5354500000
C35	30.3162000000	-32.1459000000	0.7839780000
C36	33.7562000000	-29.5518000000	0.4135590000
C37	33.9407000000	-30.9072000000	0.7284250000
C38	32.8267000000	-31.7660000000	0.8299550000
C39	31.5295000000	-31.2430000000	0.6621400000
C40	32.3105000000	-27.5609000000	-0.1341870000
C41	32.4618000000	-29.0253000000	0.2294460000
C42	31.3477000000	-29.8747000000	0.3506910000
C43	29.9467000000	-29.3198000000	0.1607730000
C44	29.8930000000	-27.9697000000	-0.5524750000
C45	30.9000000000	-26.9943000000	0.0576673000
C46	30.8059000000	-25.5965000000	-0.5519170000
C47	29.6493000000	-24.7392000000	-0.0768084000
C48	29.6351000000	-23.3042000000	-0.6526220000
C49	26.9543000000	-19.6904000000	1.6606000000
C50	28.0060000000	-19.9117000000	2.7518500000
C51	29.3366000000	-20.2759000000	2.1322700000

C52	20.4148000000	-23.0803000000	0.2613620000
C53	27.0685000000	-20.7408000000	0.5608080000
C54	28.1891000000	-21.5929000000	0.5180210000
C55	28.3358000000	-22.5517000000	-0.5005580000
C56	27.3058000000	-22.7074000000	-1.4241300000
C57	26.1864000000	-21.8717000000	-1.4072500000
C58	26.0905000000	-20.8299000000	-0.4611690000
C59	25.0672000000	-19.7349000000	-0.6539010000
C60	18.9047000000	-19.2546000000	1.5029700000
C61	22.8279000000	-18.8060000000	0.0084845900
C62	23.6922000000	-19.9109000000	-0.0527446000
C63	23.1968000000	-21.1381000000	0.3963680000
C64	21.8800000000	-21.2987000000	0.8411270000
C65	21.0396000000	-20.1652000000	0.9321530000
C66	21.5272000000	-18.9244000000	0.5096820000
H67	34.9575000000	-27.9274000000	2.2541500000
H68	36.9515000000	-26.4755000000	1.9240400000
H69	38.0877000000	-26.4008000000	-0.2960970000
H70	37.2164000000	-27.7907000000	-2.1745700000
H71	35.2194000000	-29.2397000000	-1.8308500000
H72	18.8701000000	-30.6920000000	0.7453230000
H73	23.1066000000	-27.7990000000	-0.5234870000
H74	23.0941000000	-29.2870000000	0.4533360000
H75	22.8090000000	-29.3796000000	-1.2966600000
H76	20.9469000000	-28.0150000000	0.7497070000
H77	19.3457000000	-27.8203000000	-1.1608000000
H78	20.5958000000	-28.3978000000	-2.2717600000
H79	22.0187000000	-21.8749000000	-1.3232100000

H80	22.8696000000	-24.4417000000	-3.5267100000
H81	22.1081000000	-26.8052000000	-3.2768700000
H82	19.8855000000	-25.6750000000	0.2253090000
H83	38.7547000000	-32.7900000000	1.3774500000
H84	37.4933000000	-33.5482000000	-0.5139080000
H85	38.0903000000	-31.8754000000	-0.6518890000
H86	36.0046000000	-30.6404000000	1.3320800000
H87	35.3607000000	-32.1932000000	1.7608500000
H88	33.3968000000	-33.3917000000	2.1583600000
H89	32.1664000000	-33.8384000000	0.9708490000
H90	33.8375000000	-33.6438000000	0.4575940000
H91	30.7755000000	-33.4763000000	-0.8934300000
H92	29.0598000000	-33.4837000000	-0.4137460000
H93	29.7388000000	-32.1156000000	-1.3286800000
H94	30.4897000000	-32.8999000000	1.5648200000
H95	29.4559000000	-31.5623000000	1.1381100000
H96	33.0328000000	-26.9623000000	0.4417520000
H97	32.6102000000	-27.4281000000	-1.1911700000
H98	29.4630000000	-29.2182000000	1.1498300000
H99	29.3307000000	-30.0395000000	-0.3970080000
H100	28.8729000000	-27.5628000000	-0.4975050000
H101	30.1331000000	-28.1041000000	-1.6223400000
H102	30.6887000000	-26.9173000000	1.1381800000
H103	30.7561000000	-25.6462000000	-1.6529000000
H104	31.7280000000	-25.0165000000	-0.3567720000
H105	30.0253000000	-22.4820000000	-2.3810600000
H106	30.3973000000	-22.7713000000	-0.0516300000
H107	25.3454000000	-20.5153000000	2.4177100000

H108	27.1116000000	-18.6911000000	1.2179300000
H109	27.6818000000	-20.7374000000	3.4044600000
H110	28.0880000000	-19.0116000000	3.3777700000
H111	30.1201000000	-20.4374000000	2.8824300000
H112	29.6822000000	-19.4959000000	1.4281600000
H113	19.6577000000	-23.5655000000	0.8893590000
H114	19.9249000000	-22.2354000000	-0.2488190000
H115	24.3569000000	-21.9373000000	-2.1308700000
H116	27.3860000000	-23.4553000000	-2.2130600000
H117	24.9455000000	-19.5906000000	-1.7431300000
H118	25.4772000000	-18.7867000000	-0.2810910000
H119	22.6372000000	-16.9211000000	-0.3151020000
H120	19.2953000000	-18.4973000000	2.2023400000
H121	18.7363000000	-18.7964000000	0.5152840000
H122	17.9565000000	-19.6456000000	1.8868400000
H123	23.8346000000	-22.0245000000	0.3806780000
H124	20.8939000000	-18.0381000000	0.5609670000

Structure (Figure 13 A)

E = -3183.8882117 Ha

O1	19.9181000000	-30.1398000000	-1.3299500000
O2	18.1928000000	-28.6291000000	-1.3878900000
O3	22.5337000000	-21.6724000000	-1.8590500000
O4	37.8754000000	-32.2187000000	1.0024500000
O5	34.9136000000	-32.4200000000	-1.0822500000
O6	28.5999000000	-24.9371000000	0.2618230000
O7	29.0347000000	-23.9883000000	-2.8121600000
O8	26.3069000000	-20.2759000000	2.5218300000

O9	29.5833000000	-21.9907000000	0.7715400000
O10	21.7475000000	-22.2104000000	1.3589400000
O11	25.2406000000	-20.9861000000	-2.4928100000
O12	23.7919000000	-17.1643000000	0.2995020000
O13	20.1456000000	-20.0437000000	1.7734100000
O14	12.3667000000	-28.6882000000	-4.9712800000
O15	11.8563000000	-29.0599000000	-2.8205000000
C16	16.4841000000	-30.1111000000	-4.4869200000
C17	15.0810000000	-29.5430000000	-4.7496800000
C18	17.4118000000	-29.2458000000	-3.6099000000
C19	17.3641000000	-29.5710000000	-2.1191900000
C20	21.3970000000	-28.2128000000	0.3148790000
C21	19.4888000000	-29.0121000000	-1.1366300000
C22	20.2970000000	-27.8150000000	-0.6700520000
C23	20.8567000000	-27.1241000000	-1.9408600000
C24	22.1843000000	-23.0051000000	-1.7392500000
C25	22.6921000000	-23.8745000000	-2.7114000000
C26	22.3049000000	-25.2146000000	-2.7219700000
C27	21.3959000000	-25.7201000000	-1.7738000000
C28	20.9545000000	-24.8439000000	-0.7743490000
C29	21.3277000000	-23.4900000000	-0.7246290000
C30	37.0993000000	-32.5478000000	-0.1489330000
C31	35.6341000000	-32.0962000000	-0.1373610000
C32	35.1693000000	-31.2430000000	1.0416000000
C33	32.8011000000	-32.9826000000	1.3915900000
C34	29.7466000000	-32.6192000000	-0.2080120000
C35	30.0987000000	-31.8015000000	1.0458000000
C36	33.6108000000	-29.3525000000	0.6061520000

C37	33.7729000000	-30.7032000000	0.9180400000
C38	32.6267000000	-31.5209000000	1.0764600000
C39	31.3432000000	-30.9538000000	0.8866380000
C40	32.2788000000	-27.3161000000	0.0213385000
C41	32.3517000000	-28.7795000000	0.3972790000
C42	31.2072000000	-29.5926000000	0.5040960000
C43	29.8425000000	-29.0074000000	0.2081520000
C44	29.8951000000	-27.7009000000	-0.5826630000
C45	30.8677000000	-26.7193000000	0.0747247000
C46	30.8430000000	-25.3314000000	-0.5659590000
C47	29.5038000000	-24.6132000000	-0.5056600000
C48	29.3518000000	-23.4429000000	-1.5153900000
C49	27.5729000000	-20.1493000000	1.8481800000
C50	28.6906000000	-20.7896000000	2.6790700000
C51	29.9035000000	-21.0511000000	1.8118500000
C52	20.7509000000	-22.6204000000	0.3542850000
C53	27.4481000000	-20.8137000000	0.4943850000
C54	28.4255000000	-21.7364000000	0.0722254000
C55	28.2856000000	-22.4618000000	-1.1281500000
C56	27.1680000000	-22.2245000000	-1.9206200000
C57	26.2245000000	-21.2505000000	-1.5650100000
C58	26.3652000000	-20.5050000000	-0.3711690000
C59	25.4884000000	-19.3008000000	-0.1320040000
C60	19.2550000000	-18.9320000000	1.8632600000
C61	23.2656000000	-18.3957000000	0.6175060000
C62	24.1135000000	-19.5107000000	0.4593510000
C63	23.5863000000	-20.7703000000	0.7748840000
C64	22.2476000000	-20.9408000000	1.1620100000

C65	21.4207000000	-19.8000000000	1.3417500000
C66	21.9435000000	-18.5287000000	1.0693700000
C67	14.1684000000	-29.4588000000	-3.5251000000
C68	12.7468000000	-29.0391000000	-3.8716300000
H69	16.4045000000	-31.1281000000	-4.0560200000
H70	16.9693000000	-30.2475000000	-5.4683500000
H71	14.5602000000	-28.7337000000	-2.7846000000
H72	14.1367000000	-30.4376000000	-3.0058700000
H73	14.5785000000	-30.1653000000	-5.5084400000
H74	15.1652000000	-28.5390000000	-5.2006900000
H75	17.1726000000	-28.1768000000	-3.7530400000
H76	18.4568000000	-29.3778000000	-3.9390800000
H77	17.7163000000	-30.5946000000	-1.9217800000
H78	16.3579000000	-29.4478000000	-1.6930100000
H79	22.0018000000	-27.3357000000	0.5916260000
H80	20.9723000000	-28.6459000000	1.2343700000
H81	22.0619000000	-28.9653000000	-0.1359190000
H82	19.5792000000	-27.1301000000	-0.1891710000
H83	20.0510000000	-27.0930000000	-2.7013800000
H84	21.6445000000	-27.7725000000	-2.3647800000
H85	22.3441000000	-21.2017000000	-1.0095500000
H86	23.3866000000	-23.4786000000	-3.4568800000
H87	22.6983000000	-25.8791000000	-3.4974900000
H88	20.2622000000	-25.1966000000	-0.0042284700
H89	37.6488000000	-32.8545000000	1.7135800000
H90	37.1163000000	-33.6323000000	-0.3728050000
H91	37.5892000000	-32.0442000000	-1.0019700000
H92	35.8961000000	-30.4177000000	1.1564800000

H93	35.2985000000	-31.8438000000	1.9658800000
H94	33.6625000000	-33.1556000000	2.0558400000
H95	31.9214000000	-33.4217000000	1.8814000000
H96	32.9877000000	-33.5595000000	0.4676530000
H97	28.8272000000	-33.2073000000	-0.0471066000
H98	29.5831000000	-31.9601000000	-1.0755500000
H99	30.5575000000	-33.3148000000	-0.4753700000
H100	30.2197000000	-32.4871000000	1.9009000000
H101	29.2444000000	-31.1592000000	1.3105800000
H102	34.5018000000	-28.7217000000	0.5030580000
H103	32.9632000000	-26.7358000000	0.6691390000
H104	32.6759000000	-27.1973000000	-1.0083600000
H105	29.2954000000	-28.8257000000	1.1559700000
H106	29.2329000000	-29.7371000000	-0.3496830000
H107	28.8857000000	-27.2651000000	-0.6366860000
H108	30.2235000000	-27.9033000000	-1.6210000000
H109	30.5643000000	-26.6034000000	1.1319300000
H110	31.1879000000	-25.3947000000	-1.6194300000
H111	31.5843000000	-24.6705000000	-0.0679782000
H112	29.8184000000	-24.4955000000	-3.1123300000
H113	30.3270000000	-22.9119000000	-1.5583600000
H114	26.3242000000	-19.6657000000	3.2906800000
H115	27.8016000000	-19.0685000000	1.7073900000
H116	28.3185000000	-21.7447000000	3.0859300000
H117	28.9600000000	-20.1406000000	3.5308100000
H118	30.7271000000	-21.5160000000	2.3733900000
H119	30.2774000000	-20.1173000000	1.3474000000
H120	19.9856000000	-23.1780000000	0.9131390000

H121	20.2712000000	-21.7142000000	-0.0544674000
H122	24.3323000000	-21.1057000000	-2.1137700000
H123	27.0278000000	-22.7728000000	-2.8522300000
H124	25.3471000000	-18.8048000000	-1.1115600000
H125	26.0299000000	-18.5656000000	0.4861980000
H126	23.0861000000	-16.4889000000	0.4063460000
H127	19.6047000000	-18.1926000000	2.6060700000
H128	19.1269000000	-18.4425000000	0.8817460000
H129	18.2954000000	-19.3501000000	2.1928500000
H130	24.2154000000	-21.6605000000	0.6894860000
H131	21.3328000000	-17.6322000000	1.2075800000
H132	12.3241000000	-29.3810000000	-2.0154200000

Structure (Figure 13 B)

E = -3183.9139652 Ha

O1	20.9948000000	-30.9223000000	-0.6136030000
O2	19.4361000000	-29.9190000000	0.6428340000
O3	22.1044000000	-22.3177000000	-2.0228700000
O4	37.7623000000	-32.6825000000	1.5760600000
O5	35.6515000000	-31.6617000000	-1.1029900000
O6	28.4019000000	-25.2263000000	1.6960600000
O7	29.4725000000	-24.3291000000	-1.3001800000
O8	25.3331000000	-20.4723000000	3.0789000000
O9	28.9719000000	-22.1266000000	2.1701900000
O10	21.0099000000	-22.4491000000	1.1463800000
O11	25.2465000000	-21.7751000000	-1.8826400000
O12	23.3880000000	-17.6514000000	-0.3159980000
O13	19.4067000000	-20.2548000000	0.7195610000

O14	33.6205000000	-17.4586000000	5.0160100000
O15	34.7743000000	-18.8356000000	6.4076600000
C16	31.0874000000	-18.2843000000	6.1764900000
C17	22.7745000000	-28.6622000000	-0.4128710000
C18	20.5992000000	-29.8958000000	-0.0949045000
C19	21.2620000000	-28.5293000000	-0.2473580000
C20	20.6033000000	-27.7892000000	-1.4542800000
C21	21.7494000000	-23.6255000000	-1.7979300000
C22	22.2955000000	-24.5764000000	-2.6694200000
C23	21.9343000000	-25.9214000000	-2.5614900000
C24	21.0258000000	-26.3488000000	-1.5777100000
C25	20.5143000000	-25.3846000000	-0.6973120000
C26	20.8441000000	-24.0227000000	-0.7809650000
C27	37.1099000000	-32.9217000000	0.3231780000
C28	35.9013000000	-32.0255000000	0.0483959000
C29	35.0391000000	-31.6622000000	1.2575200000
C30	32.8956000000	-33.2695000000	-0.0132511000
C31	30.4583000000	-31.9773000000	-2.2114000000
C32	30.3258000000	-31.9495000000	-0.6788380000
C33	33.4700000000	-29.7241000000	1.2357400000
C34	33.6956000000	-31.0674000000	0.9320120000
C35	32.6630000000	-31.8203000000	0.3216080000
C36	31.4369000000	-31.1827000000	0.0051028500
C37	32.1337000000	-27.6096000000	1.2696700000
C38	32.2674000000	-29.0767000000	0.9300580000
C39	31.2510000000	-29.8020000000	0.2820730000
C40	29.9649000000	-29.1030000000	-0.1027900000
C41	30.0847000000	-27.5797000000	-0.1440530000

C42	30.7143000000	-27.0448000000	1.1447600000
C43	30.7505000000	-25.5076000000	1.1978500000
C44	29.3884000000	-24.8537000000	1.0566600000
C45	29.3128000000	-23.7279000000	-0.0019528900
C46	26.7094000000	-20.3504000000	2.6709800000
C47	27.6513000000	-20.8746000000	3.7582900000
C48	29.0398000000	-21.0891000000	3.1823400000
C49	20.1431000000	-23.0504000000	0.1222570000
C50	26.8818000000	-21.1239000000	1.3845800000
C51	27.9638000000	-22.0139000000	1.2468800000
C52	28.1012000000	-22.8396000000	0.1098610000
C53	27.1338000000	-22.7594000000	-0.8850410000
C54	26.0895000000	-21.8276000000	-0.8006400000
C55	25.9784000000	-20.9559000000	0.3038380000
C56	25.0830000000	-19.7454000000	0.2158890000
C57	18.5633000000	-19.1561000000	0.3699730000
C58	22.7604000000	-18.8169000000	0.0591118000
C59	23.5882000000	-19.9201000000	0.3523060000
C60	22.9648000000	-21.1157000000	0.7348620000
C61	21.5659000000	-21.2364000000	0.7982630000
C62	20.7553000000	-20.0951000000	0.5507460000
C63	21.3653000000	-18.8919000000	0.1661780000
C64	30.7078000000	-19.1701000000	4.9800100000
C65	30.3554000000	-20.6144000000	5.3600600000
C66	30.0991000000	-21.5582000000	4.1756400000
C67	32.4378000000	-18.6109000000	6.8195900000
C68	33.6246000000	-18.2281000000	5.9673400000
H69	31.1053000000	-17.2307000000	5.8509900000

H70	30.3026000000	-18.3614000000	6.9518500000
H71	19.2793000000	-29.0192000000	1.0104500000
H72	23.2341000000	-27.6701000000	-0.5378060000
H73	23.2287000000	-29.1485000000	0.4645130000
H74	23.0080000000	-29.2768000000	-1.2959200000
H75	21.0555000000	-27.9313000000	0.6631470000
H76	19.5032000000	-27.8405000000	-1.3503200000
H77	20.8547000000	-28.3447000000	-2.3738600000
H78	21.8175000000	-21.7644000000	-1.2559400000
H79	23.0024000000	-24.2361000000	-3.4303400000
H80	22.3603000000	-26.6525000000	-3.2552100000
H81	19.7964000000	-25.6917000000	0.0731686000
H82	38.1980000000	-31.8053000000	1.5113700000
H83	36.7619000000	-33.9711000000	0.3749940000
H84	37.7984000000	-32.8399000000	-0.5374560000
H85	35.6161000000	-30.9433000000	1.8702400000
H86	34.9617000000	-32.5580000000	1.9035500000
H87	33.6664000000	-33.7219000000	0.6290280000
H88	31.9844000000	-33.8769000000	0.0988202000
H89	33.2414000000	-33.3887000000	-1.0562900000
H90	29.6218000000	-32.5300000000	-2.6711600000
H91	30.4609000000	-30.9559000000	-2.6266300000
H92	31.3989000000	-32.4569000000	-2.5218700000
H93	30.2975000000	-32.9867000000	-0.3056160000
H94	29.3512000000	-31.5150000000	-0.4053330000
H95	34.2677000000	-29.1506000000	1.7216200000
H96	32.5249000000	-27.4336000000	2.2888400000
H97	32.7998000000	-27.0316000000	0.5956210000

H98	29.1641000000	-29.3791000000	0.6136000000
H99	29.6175000000	-29.4593000000	-1.0866700000
H100	29.0796000000	-27.1480000000	-0.2882770000
H101	30.6993000000	-27.2684000000	-1.0091900000
H102	30.1024000000	-27.3994000000	1.9941200000
H103	31.4268000000	-25.1240000000	0.4147460000
H104	31.1695000000	-25.1856000000	2.1730000000
H105	28.8052000000	-25.0476000000	-1.3708900000
H106	30.2173000000	-23.1040000000	0.1228990000
H107	25.1966000000	-19.8519000000	3.8277600000
H108	26.9412000000	-19.2755000000	2.4931500000
H109	27.2611000000	-21.8337000000	4.1399200000
H110	27.6856000000	-20.1618000000	4.5996600000
H111	29.3789000000	-20.1643000000	2.6748400000
H112	19.3606000000	-23.5732000000	0.6900840000
H113	19.6557000000	-22.2423000000	-0.4470000000
H114	24.3574000000	-21.4489000000	-1.6111200000
H115	27.2072000000	-23.3710000000	-1.7856600000
H116	25.2730000000	-19.2674000000	-0.7652230000
H117	25.4092000000	-18.9964000000	0.9563020000
H118	22.7000000000	-16.9797000000	-0.5193600000
H119	18.7639000000	-18.2761000000	1.0057200000
H120	18.6834000000	-18.8829000000	-0.6931320000
H121	17.5376000000	-19.5035000000	0.5454260000
H122	23.5683000000	-21.9926000000	0.9839810000
H123	20.7649000000	-18.0006000000	-0.0351679000
H124	32.5281000000	-19.6769000000	7.0899300000
H125	32.5456000000	-18.0637000000	7.7768500000

H126	31.5269000000	-19.1612000000	4.2380600000
H127	29.8442000000	-18.6988000000	4.4752200000
H128	29.4761000000	-20.6061000000	6.0308400000
H129	31.1744000000	-21.0545000000	5.9563800000
H130	31.0366000000	-21.7104000000	3.6099600000
H131	29.7942000000	-22.5519000000	4.5497300000
H132	35.4877000000	-18.4900000000	5.8164500000
